# Environmental Stresses Induce Misfolded Protein Aggregation in Plant Cells in a Microtubule-Dependent Manner

**DOI:** 10.3390/ijms14047771

**Published:** 2013-04-10

**Authors:** Yuko Nakajima, Shunji Suzuki

**Affiliations:** Laboratory of Fruit Genetic Engineering, the Institute of Enology and Viticulture, University of Yamanashi, Kofu, Yamanashi 400-0005, Japan; E-Mail: ynakajima@yamanashi.ac.jp

**Keywords:** environmental stress, ER stress, misfolded protein, protein aggregate

## Abstract

Misfolded protein aggregation in mammalian cells is one of the cellular responses to environmental stresses. However, the aggregation of misfolded proteins in plant cells exposed to environmental stresses is still poorly understood. Here, we report the misfolded protein aggregation in plant cells in response to environmental stresses, including ultraviolet (UV) radiation, heat stress and cold stress. Treatment of grape and tobacco cultured cells with MG-132, a proteasome inhibitor, induced misfolded protein aggregation. All of the environmental stresses examined induced the endoplasmic reticulum (ER) stress response in the cells. The cells under ER stress showed aggregation of misfolded proteins. The misfolded protein aggregation was completely inhibited by treatment of the cells with trichostatin A or colchicine, suggesting that the misfolded proteins might be aggregated in plant cells in a microtubule-dependent manner. Detected aggregates were initially observed immediately after exposure to the environmental stresses (1 min after UV radiation, 5 min after heat stress exposure, and 15 min after cold stress exposure). Based on these findings, we hypothesize that environmental stresses induce misfolded protein aggregation in plant cells in a microtubule-dependent manner.

## 1. Introduction

The endoplasmic reticulum (ER) is a factory for quality control of newly synthesized proteins in eukaryotic cells. Proteins synthesized on the rough ER and imported into the ER are folded and modified in the ER. Unfolded or misfolded proteins are exported from the ER to the cytoplasm and degraded through the ubiquitin-proteasome systems [1]. Unfortunately, unfolded or misfolded proteins are accumulated in the cells under undesirable condition, and then lead the disturbance of ER homeostasis, called as ER stress. In plants, the transcription of genes related to protein folding, glycosylation, translocation, and protein degradation is activated by ER stress [2]. The enhancement of these proteins in the plant cells becomes oriented towards the maintenance of ER homeostasis to overcome ER stress. Thus, similar to mammalian cells [3–5], the ER quality control mechanism in plants is one of essential physiological responses against environmental stress [6].

We previously demonstrated that the virus-induced grapevine protein VIGG disturbs cation homeostasis in plants, which is correlated with the robustness to ER stress [7]. In plant cells under the ER stress condition, the ER quality control mechanism contributes to the maintenance of ER homeostasis and reduces the number of misfolded proteins by activating protein folding and/or degradation systems [2]. Although the ER quality control mechanism works in plant cells in response to various environmental stresses [6], the whole picture of the regulatory system of misfolded proteins in plant cells exposed to environmental stresses still remains to be elucidated. In particular, misfolded protein aggregation is poorly understood in plant cells under environmental stress conditions.

Plant viral components are transported to proteinous inclusion bodies by microtubules [8,9]. Inhibitors of microtubule polymerization suppress the formation of the inclusion bodies in virus-infected plant cells. Recently, the involvement of microtubules in the formation of the inclusions was also reported in viral pathogenesis [10]. Consistently, virus-encoded movement protein accumulates in ER-associated inclusions and along microtubules [11]. Thus, in the case of plant virus infection, microtubules may play a role in the aggregation of proteins in the cytoplasm of plant cells.

In this study, we examined protein aggregation in plant cells exposed to environmental stresses. Environmental stresses—including UV radiation, heat stress, and cold stress—induced ER stress in plant cells. Simultaneously, plant cells aggregated misfolded proteins in response to these environmental stresses. In addition, from experiments using inhibitors, we demonstrated that microtubules are essential to the misfolded protein aggregation in plant cells in response to environmental stresses.

## 2. Results

### 2.1. MG-132 Induces Misfolded Protein Aggregation in Plant Cells in a Microtubule-Dependent Manner

**Figure 1:**
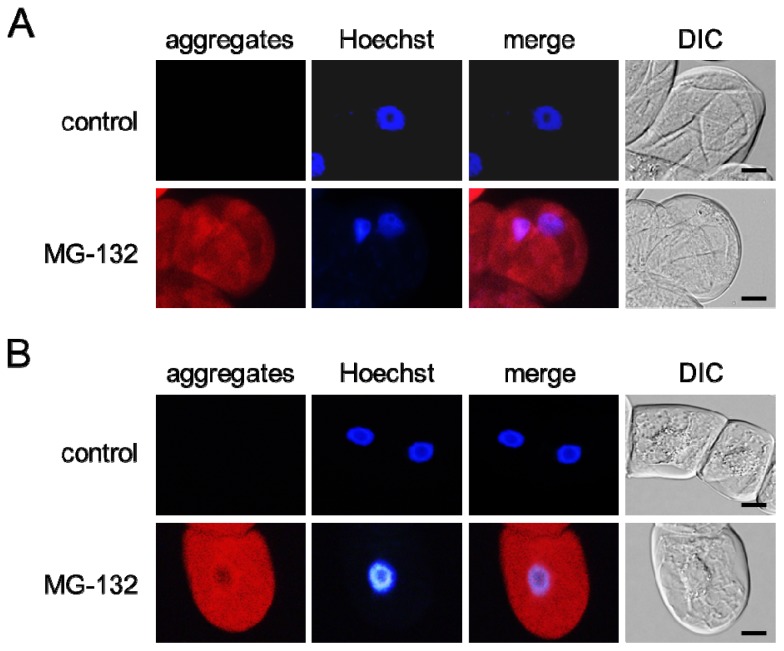
Misfolded protein aggregation in plant cells. Grape (**A**) and tobacco (**B**) cultured cells were treated with MG-132. The cells were stained with Hoechst 33342 (Hoechst) and then with a ProteoStat Aggresome Detection Kit (aggregates). The red color and the blue color indicate the fluorescence of detected aggregates and stained nuclei, respectively. Merge, a merged image. DIC, an image observed under a differential-interference-contrast (DIC) microscope. Scale bars, 20 μm.

MG-132 is a cell-permeable proteasome inhibitor that enhances misfolded protein aggregation in mammalian cells [12]. It was found that 10 μM MG-132 induced dispersed red signals in the cytoplasm of both grape (Figure 1A) and tobacco (Figure 1B) cultured cells. ProteoStat Aggresome Detection Kit we used in the present study contains a novel 488 nm excitable red fluorescent molecular rotor dye to specifically detect denatured and/or misfolded protein cargo in fixed and permeabilized cells. The kit has been used for the detection of protein aggregation in mammalian cells under pathological and physiological conditions [13,14]. Based on these reports, we judged that the red signals revealed misfolded protein aggregates in the plant cells.

**Figure 2:**
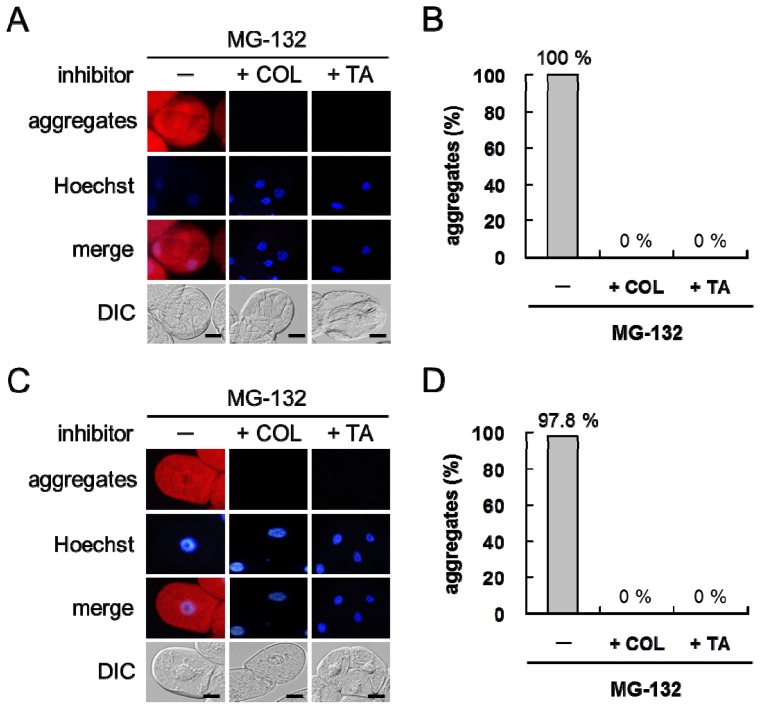
Effect of trichostatin A or colchicine treatment on misfolded protein aggregation in plant cells. (**A** and **C**) Microscopy observation; (**B** and **D**) Incidence of misfolded protein aggregation. Cotreatment with trichostatin A (TA) or colchicine (COL) and MG-132 completely suppressed misfolded protein aggregation in grape (**A** and **B**) and tobacco (**C** and **D**) cultured cells. The red color and the blue color indicate the fluorescence of detected aggregates and stained nuclei, respectively. Merge, a merged image. DIC, an image observed under a DIC microscope. Scale bars, 20 μm.

Detected aggregates were diffusely dispersed in the cells at 18 h post-treatment irrespective of cell type. MG-132 treatment led to misfolded protein aggregation in 100% of grape cultured cells and 97.8% ± 0.02% of tobacco cultured cells (Figure 2B,D). In contrast, untreated grape and tobacco cultured cells did not form the aggregates.

To determine the role of microtubules in protein aggregation in plant cells, MG-132-treated plant cells were treated with trichostatin A, a histone deacetylase inhibitor, or colchicine, an inhibitor of microtubule polymerization. Colchicine treatment depolymerized microtubules in the plant cells tested (data not shown). Trichostatin A or colchicine completely suppressed misfolded protein aggregation in grape (Figure 2A) and tobacco (Figure 2C) cultured cells. Trichostatin A or colchicine treatment did not induce misfolded protein aggregation in MG-132-treated grape (Figure 2B) and tobacco (Figure 2D) cultured cells.

Taken together, these findings suggest that plant cells form misfolded protein aggregates in a microtubule-dependent manner when the proteasome machinery is disturbed.

### 2.2. Environmental Stresses Induce ER Stress Response in Plant Cells

**Figure 3:**
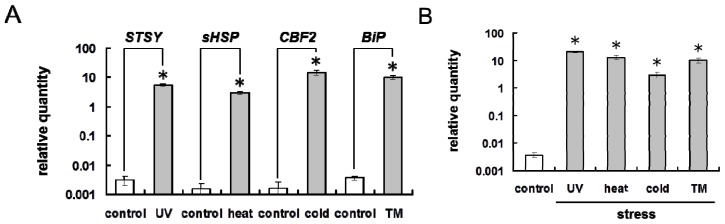
Environmental stresses induce ER stress response in plant cells. (**A**) Expression of stress marker genes in grape cultured cells exposed to environmental stresses. UV radiation, heat stress, and cold stress upregulated *STSY*, *sHSP*, and *CBF2* expression in grape cultured cells, respectively. Tunicamycin (TM) induced luminal binding protein (*BiP*) expression in grape cultured cells; (**B**) Expression of *BiP* in grape cultured cells exposed to environmental stresses. All of the environmental stresses examined upregulated *BiP* expression in grape cultured cells similarly to TM treatment. Bars indicate means ± standard errors calculated from three independent experiments. Mean values statistically different from control (*p* < 0.01) are indicated by asterisks.

To determine whether environmental stresses induce the ER stress response in plant cells, grape cultured cells were treated with UV radiation, heat stress, and cold stress; the thus treated cells abundantly expressed stress marker genes (Figure 3A). Stilbene synthase (STSY) is a key enzyme that is induced by UV radiation [7] in plant stilbenoid biosynthesis. The small heat shock protein (sHSP) is a molecular chaperone binding to denatured proteins, and is upregulated by heat stress in grapevines [15], while the CBF-like transcription factor (CBF2) is a transcriptional factor that is upregulated in response to cold stress and is related to cold tolerance in grapevines [16]. The results in Figure 3A suggested that the cells responded to each environmental stress.

Tunicamycin, which induces the ER stress response by preventing the glycosylation of glycoproteins, upregulated *BiP* (luminal-binding protein) expression in grape cultured cells (Figure 3). Because BiP proteins are master regulators of the ER stress response and act by binding to misfolded proteins [17], the upregulation of *BiP* expression reflects induction of ER stress in the cells. Similarly, each environmental stress induced the expression of *BiP* in grape cultured cells (Figure 3B). The expression levels of *BiP* in the cells exposed to the environmental stresses were equal to that in the cells treated with tunicamycin.

These findings suggest that the environmental stresses examined in this study induce the ER stress response in plant cells.

### 2.3. Environmental Stresses Induce Misfolded Protein Aggregation in Plant Cells in a Microtubule-Dependent Manner

**Figure 4:**
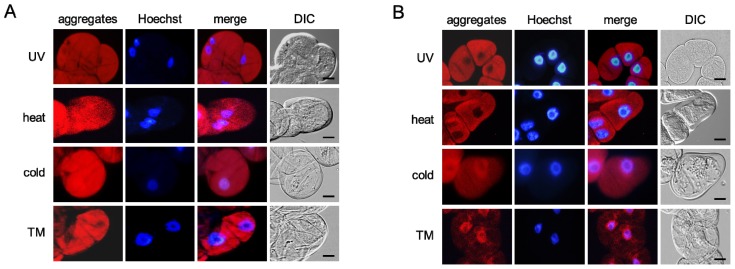
Environmental stresses induce misfolded protein aggregation in plant cells. (**A**) Grape cultured cells; (**B**) Tobacco cultured cells. The cells exposed to the environmental stresses were stained with Hoechst 33342 (Hoechst) and then with a ProteoStat Aggresome Detection Kit (aggregates). The red color and the blue color indicate the fluorescence of detected aggregates and stained nuclei, respectively. TM, tunicamycin; Merge, a merged image; DIC, an image observed under a DIC microscope; Scale bars, 20 μm.

Tunicamycin induced misfolded protein aggregation in 95.31% ± 0.02% of grape cultured cells (Figure 4A, TM) and 93.75% ± 0.06% of tobacco cultured cells (Figure 4B, TM). Likewise, UV radiation, heat stress, and cold stress induced the aggregation of misfolded proteins in both cells (Figure 4, UV radiation, heat stress, and cold stress). Detected aggregates were diffusely dispersed in the cells exposed to the environmental stresses, as similarly observed in cells treated with MG-132 and tunicamycin. The incidences of misfolded protein aggregation accelerated by the environmental stresses were as follows: 99.43% ± 0.01% in grape cultured cells and 97.08% ± 0.03% in tobacco cultured cells exposed to UV radiation, 90.53% ± 0.04% in grape cultured cells and 94.74% ± 0.05% in tobacco cultured cells exposed to heat stress, and 90.54% ± 0.03% in grape cultured cells and 100% in tobacco cultured cells exposed to cold stress.

**Figure 5:**
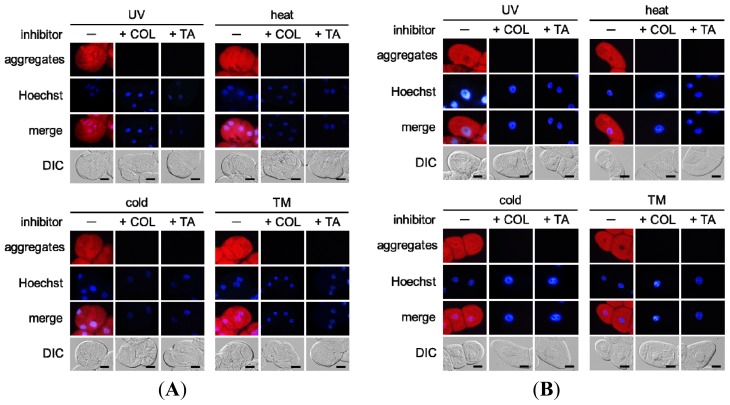
Effect of trichostatin A or colchicine treatment on misfolded protein aggregation in (**A**) grape cultured cells and (**B**) tobacco cultured cells exposed to environmental stresses. Treatment with trichostatin A (TA) or colchicine (COL) completely suppressed misfolded protein aggregation induced by the environmental stresses. The red color and the blue color indicate the fluorescence of detected aggregates and stained nuclei, respectively. TM, tunicamycin; Merge, a merged image; DIC, an image observed under a DIC microscope; Scale bars, 20 μm.

Trichostatin A or colchicine treatment abolished the aggregation of misfolded proteins in grape (Figure 5A, TM) and tobacco (Figure 5B, TM) cultured cells treated with tunicamycin. Similarly, both inhibitors completely suppressed misfolded protein aggregation in all of the grape and tobacco cultured cells exposed to the environmental stresses (Figure 5, UV radiation, heat stress, and cold stress).

Taken together, these findings suggest that environmental stresses induce misfolded protein aggregation in plant cells in a microtubule-dependent manner.

### 2.4. Aggregates Are Rapidly Accumulated

**Figure 6:**
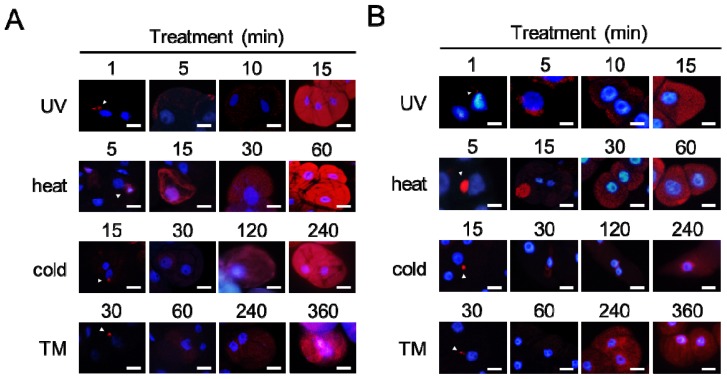
Dynamics of misfolded protein aggregation in plant cells under environmental stress conditions. (**A**) Grape cultured cells; (**B**) Tobacco cultured cells. The cells were fixed at the indicated time after exposure to environmental stresses and then stained. The images are merged images. The red color and the blue color indicate the fluorescence of detected aggregates and stained nuclei, respectively. Arrowheads indicate single or small aggregates. TM, tunicamycin; Scale bars, 20 μm.

In plant cells exposed to ER and environmental stresses, detected aggregates were observed as single spots immediately after the treatments (Figure 6). Grape (28.88 ± 0.05%) and tobacco (11.77 ± 0.09%) cultured cells showed misfolded protein aggregation 1 min after UV radiation (Figure 6, UV). Five minutes after heat stress exposure, grape (7.87 ± 0.04%) and tobacco (2.91 ± 0.02%) cultured cells formed the aggregates (Figure 6, heat). Moreover, cold stress exposure for 15 min induced misfolded protein aggregation in grape (68.55 ± 0.07%) and tobacco (20.42 ± 0.04%) cultured cells (Figure 6, cold). Similar aggregation of misfolded proteins was observed in grape (27.72 ± 0.03%) and tobacco (25.66 ± 0.02%) cultured cells 30 min after tunicamycin treatment (Figure 6, TM). After then, misfolded aggregation occurred throughout the cytoplasm of the cells irrespective of treatment.

## 3. Discussion

**Figure 7:**
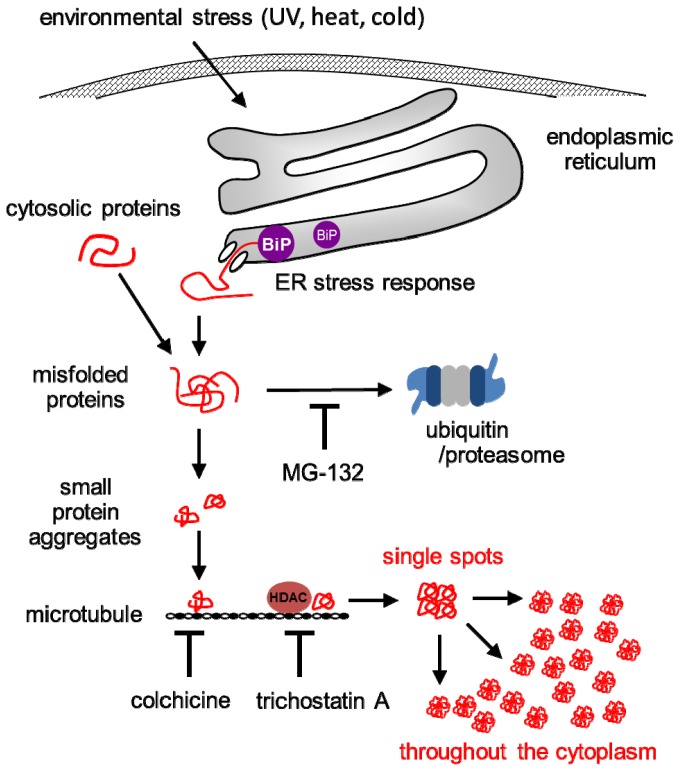
Theoretical model of misfolded protein aggregation in plant cells under environmental stress condition. Environmental stresses induce the ER stress response, including the upregulation of BiP in plant cells. Misfolded proteins are translocated from ER to the cytoplasm. Usually, misfolded proteins are degraded by proteasomes. Accordingly, misfolded protein aggregates are formed in plant cells following MG-132 treatment. Numerous misfolded proteins are likely generated in response to environmental stresses and the capacity of proteasomes to degrade them is exceeded. The aggregation of misfolded proteins occurs first in single spots and then throughout the cytoplasm. These aggregates might be formed in a microtubule-dependent manner, since colchicine and trichostatin A inhibits the aggregation. Histone deacetylase (HDAC) coordinates the formation of the aggregates on the microtubule through a yet unexplained manner(s). End lines indicate negative regulations.

A predicted model of misfolded protein aggregation in plant cells exposed to environmental stresses is shown in Figure 7. Plant cells form misfolded protein aggregates when the number of misfolded proteins exceeds the capacity of proteasomes to degrade them (Figure 1). Environmental stresses, such as UV radiation, heat stress, and cold stress, induce the ER stress response, including the upregulation of BiP in plant cells (Figure 3). Numerous misfolded proteins are likely generated in response to environmental stresses (Figure 4). Detected aggregates might be formed in a microtubule-dependent manner (Figure 5).

In mammalian cells, protein folding is interfered by environmental stresses, such as thermal stress [3], osmotic stress [4], or viral infection [5]. The interference results in the generation and accumulation of misfolded proteins in the cells. Proteinaceous inclusion body, namely, aggresome has been well studied in mammalian cells [18]. A single misfolded protein molecule forms an aggregate that is transported by microtubules to microtubule-organizing centers (MTOCs) [19]. Small dispersed aggregates are accumulated by HDAC6, a histone deacetylase, and microtubules and to form aggresomes. Finally, the aggresomes interact with various chaperones and proteases for their degradation [20]. Aggresome-like structures have been observed in plant-plant virus interactions [8,9]. The inhibition of cytoplasmic proteasomes leads to the aggregation of the potato leafroll virus 17-kDa movement protein in aggresome-like structures [8]. Microtubules are essential to the formation of these structures. Recently, the existence of aggresomal pathways by which plant cells recruit membranes and proteins into localized macromolecular assemblies in the viral infected plant cells was suggested [10]. In the present study, we demonstrated the protein aggregation in plant cells in response to environmental stresses in a microtubule-dependent manner. However, it is plausible that the aggregates formed in the plant cells under environmental stresses might be different from aggresomes.

First, the shape of detected aggregates formed in plant cells in response to environmental stresses is different from that in mammalian aggresomes. In general, one aggresome is formed per mammalian cell. Although single aggregates were frequently observed in plant cells at the early stage of misfolded protein aggregation (Figure 6), the detected aggregates were finally dispersed through the plant cells. Aggresomes form at MTOCs in mammalian cells [18], whereas plant cells do not have a single MTOC. Instead of MTOCs, several microtubule nucleation sites are dispersed at perinuclear and cortical sites in plant cells [20]. Therefore, the protein aggregation observed in the present study is not equivalent to aggresome formation.

Next, dynein doesn’t exist in higher plants. Histone deacetylase 6 binds to both polyubiquitinated misfolded proteins and dynein motors on microtubules in animal cells, and is essential for aggresome formation [21]. Although the formation of protein aggregates in plant cells by an inhibitor of histone deacetylases was suppressed (Figures 2 and 5), plant cells don’t have dynein motors on microtubules. Therefore, the protein aggregation observed in the present study might be occurred through a unique transport mechanism distinct from aggresome formation. At present, the role of histone deacetylase on protein aggregation in plant cells remains to be elucidated. Further experiments employing immunoprecipitation with antibodies to histone deacetylases would provide new information on the transport machinery of protein aggregation in a microtubule-dependent manner in plant cells.

## 4. Materials and Methods

### 4.1. Chemicals

Trichostatin A, colchicine, and tunicamycin were purchased from Wako (Tokyo, Japan). MG-132 and Hoechst 33342 were obtained from Enzo Life Sciences International (Plymouth Meeting, PA, USA).

### 4.2. Plant Materials

The tobacco BY-2 cell line was obtained from RIKEN BioResource Center. Tobacco cell suspensions were maintained in modified Linsmaier and Skoog medium, as described by Nagata *et al.*[22]. As described previously [23], grapevine cultured cells prepared from meristems of *Vitis vinifera* cv. Koshu were used. Grapevine cell suspensions were maintained in modified Gamborg’s B5 medium at 27 °C.

### 4.3. Chemical Treatment

MG-132, a proteasome inhibitor, was added to the cell suspensions (final concentration, 10 μM). The cell suspensions were incubated at 27 °C for 18 h and then subjected to the staining of misfolded proteins. Trichostatin A or colchicine was simultaneously added to the cell suspension treated with MG-132 or exposed to environmental stresses. The cell suspensions were incubated at 27 °C for 18 h and subjected to the staining of misfolded proteins.

### 4.4. Stress Treatment

One milliliter of a tobacco or grapevine cell suspension was added into each well of a 24-well plate. For ultraviolet (UV) stress treatment, the cell suspension was exposed to UV-C light (254 nm) for 15 min at room temperature. For heat and cold stress treatments, cell suspensions were incubated at 45 °C for 1 h and 4 °C for 4 h, respectively. For ER stress induction, 1 μL of 5 mM tunicamycin (final concentration, approximately 5 μM) was added to the cell suspension. The cell suspension was incubated at 27 °C for 6 h. After each stress treatment, the cell suspension was subjected to the staining of misfolded proteins or RNA isolation.

### 4.5. Staining of Misfolded Proteins

The cells were fixed with 4% paraformaldehyde and then stained with Hoechst 33342 and a ProteoStat Aggresome Detection Kit (Enzo Life Sciences International) in accordance with the manufacturer’s instructions. After staining, the cells were analyzed under a fluorescence microscope (Olympus, Melville, NY, USA). DP Manager software (Olympus) and Windows Photo Gallery (Microsoft, Redmond, WA, USA) were used for the correction and adjustment of the brightness and contrast of photographs. The incidence of misfolded protein aggregation was quantified manually by counting the number of cells positively or negatively stained for the misfolded protein aggregates. This quantification was conducted in five independent experiments.

### 4.6. RNA Isolation

The cell suspension was filtered using a piece of filter paper (Advantec, Tokyo, Japan). The cells on the filter paper were quickly transferred into a mortar containing liquid nitrogen and homogenized with a pestle. RNA isolation was performed using RNAiso Plus (Takara, Otsu, Japan) in accordance with the manufacturer’s instructions. Briefly, RNAiso Plus solution was added to the cell powder. The mixture was incubated for 5 min at room temperature and then centrifuged at 15,000 rpm for 5 min at 4 °C. The supernatant was collected into a new microtube and treated with chloroform. After centrifugation at 15,000 rpm for 15 min at 4 °C, an equal volume of isopropanol was added to the supernatant. After centrifugation at 15,000 rpm for 10 min at 4 °C, the pellet was recovered and then dissolved in distilled water.

### 4.7. Quantitative RT-PCR Analysis

First strand cDNA was prepared from total RNA using a Prime Script RT Reagent Kit with gDNA Eraser (Takara) in accordance with the manufacturer’s instructions. Quantitative RT-PCR was performed using the Thermal Cycler Dice Real Time System (Takara) with SYBR Premix Ex Taq II (Takara). PCR amplification was performed for 40 cycles at 95 °C for 5 s and 60 °C for 1 min after an initial denaturation for 30 s at 95 °C. β-Actin was used for normalization, and the expression level of each gene was expressed as a relative value. The nucleotide sequences of the primers used were as follows: *STSY* primers (5′-AGAGAATAATGCAGGAGCACGA-3′ and 5′-GCTGACCCATCGCCAAA-3′, corresponding to bases 903-924 and 1028-1012 of *V. vinifera* stilbene synthase 1 gene, GenBank ID: DQ987603, respectively), *sHSP* primers (5′-ATTCACCAGCGGTGCTCTATC-3′ and 5′-TCCACCTTCACT TCCTCTTTCTTT-3′, corresponding to bases 78-98 and 215-192 of the *V. vinifera* small heat shock protein, 17.1 kDa mRNA, GenBank ID: GU169699, respectively), *CBF2* primers (5′-CGCTGC TTCTTCCGACTCTC-3′ and 5′-CACTCACCCATTTGTTCTCATTTC-3′, corresponding to bases 103-122 and 239-216 of the *V. vinifera* CBF-like transcription factor (Cbf2) gene, GenBank ID: AY390376, respectively), *BiP* primers (5′-CTCGCATTCCCAAGATCCA-3′ and 5′-TCCAGT TTCTTCCCCTCCTTC-3′, corresponding to bases 1106-1124 and 1251-1231 of the *V. vinifera* putative luminal-binding protein, GenBank ID: JQ713556, respectively), and β-actin primers (5′-CAAG AGCTGGAAACTGCAAAGA-3′ and 5′-AATGAGAGATGGCTGGAAGAGG-3′, corresponding to bases 409-430 and 537-516 of *V. vinifera* β-actin, GenBank ID: AF369524, respectively).

### 4.8. Statistical Analysis

ANOVA and Dunnett’s multiple range test using Excel statistics software ver. 2012 (Social Survey Research Information, Tokyo, Japan) were used to assess the significance of differences among stress treatments.

## 5. Conclusion

In conclusion, we detected misfolded protein aggregation in plant cells exposed to environmental stresses. These data may support the novel concept that environmental stresses interfere with protein folding and induce misfolded protein aggregation. Detected aggregates might be formed in plant cells in a microtubule-dependent manner. One important issue to be clarified is the function of detected aggregates in plant cells exposed to environmental stresses. Most likely, detected aggregates in plant cells may be an alternative system for the degradation of misfolded proteins generated by environmental stresses, when the degradation capacity of the proteasomes is exceeded. Further studies are under way to determine the role of detected aggregates in plant cells exposed to environmental stresses.
